# Even we are important: Sexuality and the degenderisation of people with disabilities in the linguistic landscapes of two South African universities in the Western Cape province

**DOI:** 10.4102/ajod.v8i0.568

**Published:** 2019-11-22

**Authors:** Temitope O. Adekunle, Gift Mheta, Maleshoane Rapeane-Mathonsi

**Affiliations:** 1 Department of Media, Language and Communication, Faculty of Arts and Design, Durban University of Technology, Durban, South Africa

**Keywords:** linguistic landscaping, critical discourse analysis, multimodality, sexual orientation, South African universities, interpretive paradigm, degenderisation, people with disabilities, signs, images, texts, billboards, posters

## Abstract

**Background:**

This study focuses on the positioning of gender, sexual orientation and people with disabilities in the linguistic landscapes of two selected South African universities, which are located in the Western Cape province.

**Objectives:**

This study aims to answer the question: How are power relations depicted through linguistic landscaping in the universities?

**Methods:**

Given that there is minimal empirical data in this field, the researcher approached this question by exploring the way in which sexual orientation and people with disabilities are perceived, via the modal resources used in the categorisation of toilet users at the institutions. Specifically, toilet signage was observed as there were only a few other signage or forms of support (such as ramps and lifts – some of which may seem disability-unfriendly in terms of space) and acknowledgement in other places at the institutions for people with disabilities. Data (signs, images, texts, billboards and posters) were collected by means of photography. The interpretive paradigm was used to determine the choice of methodology: critical discourse analysis and multimodality. These were also used to thematically analyse the collected data.

**Results:**

Findings revealed that sexuality, as well as subtle inequality, unfortunately remain unravelled areas in South Africa’s higher institutions of learning. In addition, the degenderisation of people with disabilities appears to be prevalent at the institutions, although this may not necessarily be reflective of practices at all higher education institutions in South Africa.

**Conclusion:**

Nonetheless, the examined results are stimulating indicators of hegemonic and preferred practices in public places. They also depict the obtainable dissimilar scales and imbalances in society, which are not addressed may impede other authentic and ongoing measures of social integration and advancement.

## Introduction

Linguistic landscape (LL) is a recently researched branch of sociolinguistics. It was termed ‘gestalt’ by Ben-Rafael (2009:43), which connotes the understanding of different concepts that make a standard or well-controlled phenomenon. It is birthed from the perception that texts are historically, socially and politically significant communication tools (McGregor 2010). Linguistic landscapes are texts that are publicly displayed, and which mostly provide some understanding of advocated and embraced (Ben-Rafael 2009) beliefs, ideologies and religions, among others. They are thus termed ‘visible languages’ (Bourhis & Landry 2002) because they enhance an understanding of the communicative values of texts and their contextual influences. Dagenais et al. (2009) thus assert that language serves two functions: namely, symbolic and informative; that is, the nature of the conveyed message and the type of language being used in public spaces (Kotze 2010). The ‘symbolic’ functions of linguistic landscaping comprise semantic interpretation of cultural relationships, uniqueness, linguistic prestige and power dynamics while the ‘informational’ functions focus on creating awareness and informing the audience about some phenomenon. It also provides adequate knowledge of the functional business of linguistics and discourse in an organisation and society at large and, more importantly, the intended meanings of texts alongside all forms of influence (political, economic and historical, among others) on linguistic landscaping. The prevalence of power dynamics is, therefore, of significance in this study and is explored via the identification of gaps in language use and practice at the selected universities (which are called ‘University A’ and ‘University B’ in this article) in relation to sexuality, gender identity and the inclusion of people with disabilities.

Most studies on LL focus mainly on discourses of power, space and language. This research also approaches the collected data with due consideration of these factors, as well as in terms of understanding the utilisation of modal resources in conveying meaning to a target audience. In addition, discussions were based on the discovery of the different weightings of the services provided to the staff and students (as well as other users) of the universities, while also considering the possible impact of inequality on identity. Power relations and standards were noted in relation to the accommodation or possible perceptions of people with disabilities, varied gender identities and sexual orientations. This study highlights the important matter of social inclusion or exclusion given the human rights culture in which we are currently immersed. Social inclusion manifests in many forms, and people can feel excluded or welcomed in various ways, including through media representation or the lack thereof.

McMullan (2008) claims that about 15% of people in the world have disabilities in various forms. Approximately 80% of these are from developing countries, with a higher percentage from the Asia-Pacific region (McMullan 2008). Regardless, people with disabilities are treated collectively as though they have no gender or sexual orientation (Women with disabilities Australia n.d.). Meanwhile, from the gender perspective, approximately 12% females and 19.2% males have some form of disability (World Health Organization [WHO] and the World Bank 2011). As a result of the genderless and collective treatments, most people with disabilities are generally ostracised and are not included in society (Research brief on disability and equality in South Africa 2017:1). Therefore, this article focuses on the various ways by which integration and accommodation are enhanced (or not) by the presented modal resources at the selected universities. The focus is, among other things, on the integration and accommodation of sexual differences, gender identities and students with disabilities by the facilities (in this study for instance, observed toilet spaces) accorded to them. Additionally, the researcher identified elements of discursive silence in the process of the research, which facilitated a broader analysis of some identified power dynamics, relevance of time and space as well as their impact on the LLs of the universities in terms of the indicated phenomena (sexual or gender identities and the degenderisation of people with disabilities) and the constituents of the learning space. These will be explored in detail in the following sections.

## Literature review

This section presents reviews on LLs in relation to sexual orientation, gender identities and people with disabilities in the selected South African universities. Linguistic landscape is also termed ‘semiotic landscape’ as meanings are expectedly tied to signs (Jaworski & Thurlow 2010) and these meanings are supposed to be understandable to those who occupy that space. Space is thus sociolinguistically depicted in that it is easily detected and accepted (Lefebvre 1991) and represents policymakers’ and authors’ views and perceptions (Trumper-Hecht 2009) about social, political, historical and economic issues. Lefebvre (2009) explains that social and political spaces are genuine and effective as the maintenance of power and dominance bind them. In other words, languages used in public places are significantly influenced and preserved (or not) by certain levels of power dynamics. Blackwood and Tufi (2012) for instance examined the influence of LL on French and Italian Mediterranean coastline cities, where French is the compulsory language used (aimed towards marketing products), accompanied by other languages which the manufacturer of those products chooses. This indicates that authors may have a strong linguistic influence on signs, which may also be controlled by or for economic reasons. An implication of this is the daunting impact of controlled or authorship dependency on produced texts. Authors are then likely to be deeply or personally absorbed in their creations or doing it professionally for many reasons, for instance, economic reasons. This indicates that they may either be objective or subjective, depending on the situation. Most importantly, authors are significant individuals who influence LLs, as well as their placements in public places.

In addition, the displayed language code or text is as significant as its placement in public spaces. Sign placement is key in an attempt to make a sign visible to the target audience. Backhaus (2007) studied the LL of train stations in Tokyo, where he distinguished between languages used in public and private places, as well as the placement of those signs. This confirms that any discourse that includes the use of audio, visuals and even humans (Shohamy & Waksman 2009) and is positioned in a place that is visible (and meaningful) to all, can be referred to as LL. These actions are purposeful and there is an intention to convey a message with any utilised symbol, text or sign within the social and/or cultural space. The intensified interest in linguistic landscaping is based on the premise that besides the use of language, publicly displayed signs or symbols also reflect multicultural and multilingual relationships (Backhaus 2007). These texts and symbols are known as modal resources that represent existing, available as well as used and unused linguistic and cultural tenets in society. That is, language, alongside other semiotic resources, can mirror society as well as the applications and occurrences in that given space. Symbols also indicate an inner process of awareness that links people to causes and events (Langdridge 2007) and they echo circumstances and perceptions (Creswell 2007). Images disclose certain categories of awareness and signification (Prosser & Loxley 2008), which help in obtaining in-depth details about both the space and its occupants.

Linguistic landscapes as being socially and meaningfully shaped is also emphasised by Shohamy and Waksman (2009) who express that written texts and signs are amendable and can be re-shaped and re-positioned as it suits the authors of the signs and in some instances, the occupiers of those spaces. This reveals the dynamicity of publicly spaced signs in addition to the capability of the spaces to enable all sorts of mediation and competition. Nonetheless, it is believed that the modal resources that are used in public spaces may not speak to the linguistic and cultural diversity (Moletsane, Hemson & Muthukrishna 2004:61) of the selected spaces. Researching publicly displayed forms of communication in diversely populated academic communities is thus critical because diversity is a controversial issue that may lead to chaos and disunity if not duly considered and managed. Linguistic landscapes’ recently observation by researchers stems from the conviction that a community’s language usage reflects in the texts displayed in public places (Shohamy & Gorter 2009). Linguistic landscapes present the prominence and importance of language via their display (as well as how they are displayed) on posters, billboards and street poles. An investigation into linguistic landscaping enables the comprehension of the nature of the existential relationship between language and society (Mpendukana 2014), as well as the role one plays in affecting the other. Hence, the exploration of LLs in multilingual contexts (Coulmas 2009). This accentuates the importance of the LL as the society’s lingual mirror, which detects monolingualism, bilingualism and multilingualism. Therefore, research in such an area has a significant impact on sociolinguistics.

For this research, diversity is a means of engaging with cultural, racial and ethnic differences in order to attain unity (Gilligan 2002:9). It is a process of dissimilarity that is dictated by various social dynamics (Goduka 1996:68) that have been, according to Cross and Naidoo (2012), birthed, constructed and supported by humans as well as society. This is the case with many higher institutions. For instance, students who are admitted into universities display the hugely diverse nature of the learning community in terms of race, ethnicity, nationality, culture, sexuality, gender identity, religion, language, physical ability or disability or historical and political affiliations, among others. Therefore, the power of publicly displayed texts and information cannot be overemphasised in today’s academic institutions, where posters and symbols are used to inform students and/or staff about products and services.

### Sexuality, sexual orientation and gender

While sexuality deals with people’s expressive and experiential knowledge sexually (such as views, feelings and interest in and for other people), sexual orientation is one’s sense of uniqueness about sexuality in a society that has diverse sexual distinctiveness (Weisgram & Bigler 2007:266). Foucault sees sexuality as a crucial aspect of the political tussle, which also extends into the individually motivated struggle for supremacy (Sawicki 1986). His popularity among feminist theorists stems from the fact that he disassembles prevailing but concealed forms of hegemony (Diamond & Quinby 1988). Sexuality is a form of passionate or sexual fascination towards someone of the opposite sex or gender (heterosexual), same sex (homosexual), both sexes (bisexual) and not being attracted to anyone (asexual) (Reiter 1989:139–150). Causative factors of these differences are usually a result of genes, hormones and other forms of societal motivations.

Nonetheless, as a result of diverse conventional social and cultural orientations and viewpoints, Nittle (2012:5) explains that anything other than heterosexuality is not normally considered natural. Conventional views on gender usually develop into universally accepted notions that exert influence over the people living in a community (Koblitz 2005:110). This encourages stigma and stereotyping (American Psychological Association 2006:25) in societies. Stereotyping is any publicly known or acquired belief about a group of people, which includes a clustered perception of diverse aspects of their lives such as cultures, history, capabilities, gender and occupation and politics, among others (Brian 2013:11). Hence, there are popular beliefs and expectations about gender, and these are instituted by societal standards.

This confirms Foucault’s (1976:77–91) assertion that power and societal structures and concepts influence people. Foucault (1976:1–14) states that power does not only subdue or inhibit phenomena; it also produces them. To him, sexuality is too essential to be suppressed, because any kind of suppression also impacts related discourses, as they are intentional systems which may be sometimes double-sided. He explains that although sexuality is expelled and denounced, it also gets discussed and remains reachable (Foucault 1976:92–102). This article will however not focus on Foucault’s stance on sexuality as some of his views on sexuality have been widely criticised as not efficiently deliberating on the influence of human aspirations on sexual standards and ethics.

Brian (2013:11) defines gender as the informally created roles, behaviours, actions and qualities ascribed as the appropriate norms for men and women. Once an action or view about gender identity or classification does not conform to this perception, there is then cause for stereotyping of all sorts. Gender stereotyping is a one-dimensional simplification of people’s capabilities, performance and roles according to their gender grouping (Yasemin 2008). It is a worldwide phenomenon that has for years been entrenched around the globe. This has also found its way into diverse spheres of life, including higher educational systems whose purpose from inception was the creation of gender equality (Bailey 2003:5).

A result of action taken by the Southern African Development Community (SADC) in eradicating stereotypes associated with gender was the endorsement of gender equality in education (UNICEF 2013:30), as well as in the national region. Moreover, the South African government has for some time been in a persistent struggle to eradicate gender stereotyping in education, with various policies focusing on addressing this level of stereotype passed soon after 1994 (Chisholm & September 2005:24). However, with regard to the LGBTQIA (Lesbian, Gay, Bisexual, Transsexual, Intersexual, Asexual – all categorised as Queer) community, the situation is more complex for South Africa (Kings 2014:67). The apartheid government was outrightly unaccepting of Lesbian, Gay, Bisexual, Trans and Intersex (LGBTI) communities. Such communities were placed under the *Sodomy Acts* and it was a 7-year jailable offence if found guilty (George 2014:34). However, the end of the apartheid government ushered in the end of the *Common Laws Act* that bound the LGBTI communities as the new constitution banned any sexuality-related discrimination (in Section 9[3]) (Kings 2014:67).

This may have been a liberating event for the community as studies, including those of Kings (2014:19) and Ansary and Babaii (2003) reveal the presence of diverse sexuality among South African learners. Brian (2013:12) thus states that instead of society influencing people’s gender or sexual status, an individual should be allowed to determine his or her sexuality. Tuwor and Sassou (2008:365) argue that schools must be gender-sensitive and empowering for gender equality to take place. They explain that there must be a balance of interests and support for each gender identity in an enabling environment where they are well-represented and accommodated. This could be achieved without placing harmful scrutiny on learners’ sexuality (Elkins & King 2006:13), with other life-altering issues (such as academic, social success) left unhandled. That is, just as it is crucial that practicality is evident in language planning and policies such that what is advocated (equality) is indeed practised by all and sundry (Prinsloo 2011), the policy of social equity should be more pronounced than it currently is (Cross 2004:389). Therefore, studies that examine language policy and social equity are important, so that detected inequalities are scrutinised, and all social groups and identities gain equal status based on the findings of such studies.

### Gender, sexuality and disability issues

Gender is one of the most significant classifications of social administration (Committee on the Elimination of Discrimination against Women – CEDAW 1988), and it is represented in all humans – the physically abled and people with disabilities alike. Interestingly, it implies that both males and females with disabilities have similar experiences of disability, thus their classification and perceptions in society (CEDAW 1989). This has led to the variously observed forms of disadvantages in terms of how people with disabilities are treated, which is most often reproduced in their experiences, most of which are also inconsiderate of their gender fluidity.

Different things often perpetuate the sole acceptance of heterosexuality in society, one of which is the concept of hegemonic masculinity which is also linked to manliness, potency and effectiveness (Jewkes & Morrell 2010). For instance, males are classified as boys or men who should generally be manly or have a macho physique (Devor 2009:13) and be assertive, with females classified as girls or women, who should generally be fragile, pretty and gentle (these notions are currently being combatted). This is the reverse and irony of perceived and actual masculinity (Cheng 2009; Shakespeare 1999) and sexual orientation, as well as the realities of the needs of people with disabilities. There have been various attempts to align sexuality and studies related to disability (Garland-Thomson 2002). Sexual and disability identities and challenges have been argued to possess strikingly similar traits, especially in terms of controversial theories of supremacy (Steyn & Van Zyl 2009) for the former and the theory of able-ism and suppression of people with disabilities (Campbell 2013) for the latter. Some of Campbell’s (2013) gender and disability similarity categorisations are provided in Table 1:

**TABLE 1:** Similarities between sexuality and disability stances and theories.

Queer	Disability
Stereotypes and discrimination	Stereotypes and discrimination
Gender or sex binary	Impairment or disability binary
Aversions to the heteronormative movement	Aversions to being equated with able-bodied counterparts
Protests for improved acceptance and accommodation	Protests for improved acceptance and accommodation

*Source*: Campbell, F.K., 2013, ‘Re-cognising disability: Cross-examining social inclusion through the prism of queer anti-sociality’, *Jindal Global Law Review* 4(2), 209–238.

While available data may be scarce, it provides insight into disability (Taylor 2001:15–33) and its stance in South Africa. An estimated 5% of South Africans were living with disabilities as of 2001 (Appunni, Blignaut & Lougues 2013). This percentage gives a somewhat fair idea of the population of people with disabilities who are residents of South Africa. The *Promotion of Equality and Prevention of Unfair Discrimination Act 4* of 2000 (PEPUDA 2000) is listed under Section 9(4) in the South African Constitution that acknowledges the significance of scrutinising discrimination and the obliteration of social and economic imbalances (National laws on labour, social security and related human rights n.d.: 7). The ninth section of this act proscribes unfair discrimination (Research brief on disability and equality in South Africa 2017:7) of any sort, namely:

- denying people with disabilities the relevant facilities and support that could enhance their effectiveness in society
- breaching the conventions of the South African Bureau of Standards in terms of environmental convenience
- failure to remove hurdles that may prevent people with disabilities from having equal rights or failure to ensure that they are sufficiently accommodated in society.

Therefore, it is important to continuously study the power balances in society so that equal measures are used when dealing with diverse gender or sexual identities and the abilities and functionality of people. Linguistic landscaping can then be said to be a powerful display of strength (Papen 2012) – linguistically, economically and socially.

## Methodology

A qualitative methodology was utilised in this study, underpinned by the interpretive paradigm which focused on an understanding of the observed multimodal resources on the selected campuses of the universities. A case study design was employed as the researcher visited the selected sites and purposively collected data that were based on sight (via the means of photography). The collected data were thereafter analysed using multimodality discourse analysis (MDA) and critical discourse analysis (CDA) in order to effectively manage diverse kinds of texts, graphics and images. These were also the study’s theoretical frameworks. Data units were purposively collected over a 3-day period. Of Denzin’s (2006) categorisation of four types of triangulation, the investigator (examining and interpretation of data by the researcher and two supervisors) and theoretical triangulation (the use of multiple theories) were applied to enhance the interpretation of findings for this study.

Multimodality discourse analysis is a communication theory that is absorbed in semiotics (Murray 2013:36). It enables an adequate description of the resources that are used to communicate ideas (via writing, visual drawings, cartoons, images, etc.) or other kinds of information. Critical discourse analysis, on the other hand, is an interdisciplinary method of studying discourse that perceives language as a shared process (Fairclough & Holes 1995:47). Discourse analysis helps in understanding social relations (Fulcher 2010:7) as it is a lucid approach of analysing and interpreting (McGregor 2010:2) the world via semiotic resources. Fairclough (1992:135) explains that CDA scientifically researches both the hidden and obvious causes and effects of a text. It helps explore the portrayed power dynamics within texts. Its aim is not to provide explicit answers to issues, but rather to enable ontological and epistemological queries (Olson 2007:29). Critical discourse analysis is thus considered a standardiser of social structures and interactions (Wodak & Meyer 2008) in a way that is equally beneficial for all who live in the society. Critical discourse analysis can also create positive reinforcement if it impacts on society’s use of language and semiotic means of communication (McGregor 2010:2). The research does not aim to critique theoretical standings, but rather utilise the chosen theories or methods (which were also the methods of data collection and analysis) in data collection and analysis. Critical discourse analysis and MDA guided the structure of the research, data collection methods, interpretation and analysis of data on both clear and hidden patterns.

The selected universities were traditional higher institutions in the Western Cape province. A campus each for both universities was visited by the researcher once permission was granted. This was deliberate as it was anticipated that, being in the same location, most or all of their policies (especially regarding students) would be similar and be in sync with that of the national government. Existing institutional policies (language, disability and sexual orientation), which were all-encompassing and acknowledging of diversity, were also considered in the selection of the institutions. An in-depth qualitative method was utilised in exploring the displayed texts on both selected campuses. The researcher used a digital camera and phone camera to take pictures of intra-campus signage. Collected data also included other semiotic resources such as brochures, website information and marketing profiles that were downloaded from the universities’ websites. A total of 400 data units were collected on both campuses (200 on each campus) as they were deemed suitable for the nature and questions of the research. Not all the collected data were eventually used as some pictures were blurred and had to be deleted from the lot. Hence, 200 data units were interpreted and analysed. A handful of the purposively selected data remarkably pointed towards the issue of hegemony with regard to the degenderisation of people with disabilities and perceptions about sexual orientation (currently with minimal empirical data), which the researcher is of the opinion requires unremitting critical engagement. Some of the selection criteria were: language or signage used, displayed ideology and the place(s) of placement. These enhanced an understanding of distinctions and connections between data; the use of space and the intended audience; presentation of ideals as well as the benefits of the utilised signage.

Examining the textual and contextual interactions of texts gained much consideration during analysis, as Rogers et al. (2005) established the importance of the description and interpretation of the establishment and alteration of social practices through data. Data were interpreted and analysed thematically. Themes were categorised as suggested by McGregor (2010) and Van Dijk (2006). Occurrence and recurrence of themes were also noted and scrutinised. Themes were coded (Chamaz 2006) and reviewed to examine signage used and the possibilities of existing power dynamics in texts (Lucke 1996). The researcher used these steps as they resonated with Gorter’s (2006) profitable coding ideology, which postulates the prioritising of certain steps before, during and after data collection and analysis.

### Ethical considerations

This university-specific (Durban University of Technology’s) ethical consideration was complied with during the course of the research, as there was no risk to humans, environment, a sensitive research area or any need for contact with animals.

## Findings and discussion

### Gender signage

**FIGURE 1:**
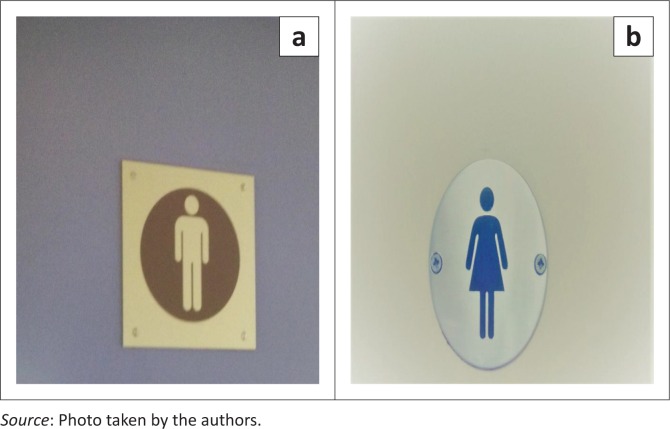
(a, b) Toilet sections for the able-bodied.

Amidst diverse modes of communication, advertisements and awareness raising found on the LLs of the universities were those on the toilets, which became significant in this study. Each sign on the doors connotes the designated toilet spaces for identified genders (male and female – as in Figure 1a and 1b). Signage is a confined speech act that occurs or is placed at a public or private site (Kallen 2009:270). As seen below, two sections of the toilets had commonly used logos for the male and female toilets at both universities.

**FIGURE 2:**
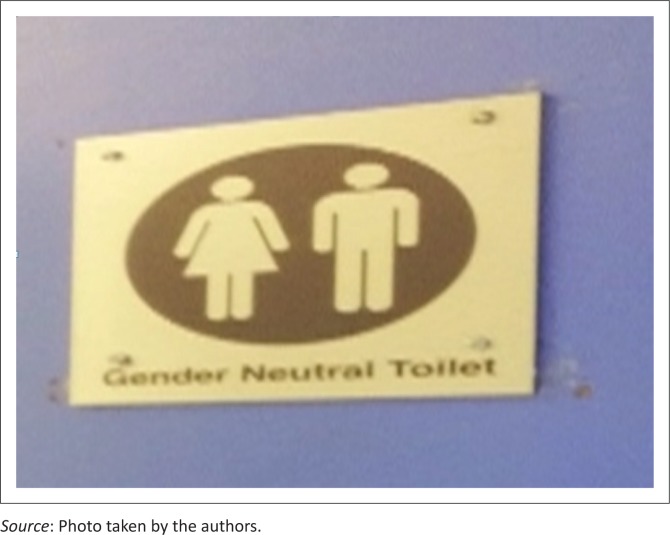
Toilet sections for gender-neutral persons.

However, University A has another toilet space which is labelled ‘gender neutral’ (see Figure 2).

### Implications of the utilised signage

One would think that the placement of such signs at the university is a move in the right direction, considering Nittle’s (2012:5) explanation that the generally or conventionally perceived norm is that anything other than heterosexuality is not considered normal. Hence, the movement of the university towards embracing students’ choices may be a welcome development. However, acknowledging that not everyone wants to be identified as a male or female may not be enough in the strive for gender equality because there is a level of silence in the conveyance of this message. Such labelling may fuel controversies about the classification and identity of ‘gender-neutral’ persons and whether the term should even exist. One also ponders on the safety of such spaces for users if the term is wrongly or inappropriately construed.

In addition, having a gender-neutral space could then possibly be a move by the management to encourage space users’ individual preference; in that homosexuals, bisexuals and asexuals, among others, can make an informed decision about what space to use without any form of discrimination or jesting. This raises doubts, however, as to whether this separation could really solve problems relating to sexual differences or add to them, as well as whether the usage of such linguistic code helps to show an acknowledgement and/or appreciation of sexual differences or actually leads to the invasion of people’s privacy (whether or not they want to be seen in that light), thus creating a further divide.

The provision of a toilet space for this group also suggests further information:

- There is no space on the campus and people of this gender identity (regardless of their population and location on campus) should manage with the provided space.
- There is an acknowledgement of such gender diversities and decisions are in motion to create more spaces to accommodate users, among others.

Either way, there may be underlying power dynamics that make a group more significant and in a position to be more urgently and fairly treated than another (Foucault 1976).

### Discursive silence

Strikingly, despite the position of South Africa on sexuality, collected data indicate that University B appears mum on the issue of sexuality, as there was no sign that signified an awareness of sexual difference or the presence of different sexual groups on campus. This confirms Dayan and Katz’s (1992) assertion that linguistically, some texts are distinguished with fixed discourses or silence. This may be a way of publicising a pivotal perspective, history or action. It may also be aimed at ensuring that audiences reflect on or absorb situations in order that transformations or alterations occur, bad to good, and vice versa. Silence is either an absence of meaning or a marker of the beginning or end of an utterance (Saville-Troike 1985:3–18). University A, on the other hand, barely showed an awareness of gender identities on one of the toilets by using the term ‘gender-neutral’. As this signage was sighted once on only one of the toilets on the Upper Campus, it may, however, not be interpreted as an absolute awareness or acceptance of the phenomenon. It affirms Pavlenko’s (2009:247) classification of LL as ‘the expression of language conflicts’. Silence here is thus implied by the authors’ deliberate (or not) decisions to be more vocal about some issues than others. Such LLs are likely to influence signage, their usage and placement (location and arrangement), as well as the implied and conveyed message.

Such silence indicates that these types of issues are either not perceived as problematic or, apparently, people have given up and prefer to be silent because of fear and other reasons, or they are simply indifferent to those issues, hence consenting to the Yemeni proverb that ‘if speech is of silver, silence is golden’. This confirms one of the unique characteristics of Foucault’s analysis in his conceptualisation of power. Foucault claims that power operates more efficiently when it is implemented through dynamic constrictions, which then invoke a limiting reaction from subjects (Tremain 2002). More so if the absence of English on signage renders it less modern (Lee 2006) or incomplete and the setting less-developed or devoid of globalisation, an unequal representation of space users in a space renders certain un/misrepresented concepts insignificant and less popular. Likewise, this finding may be one of the loopholes of studies that focus on gender identity or sexual orientation issues in South Africa and around the globe, as research astutely reveals the presence of varied sexualities among South African learners (Kings 2014:19). Texts are connected and observing what is said or unsaid (given) (Fairclough 2003:40) is crucial in the study of texts. Textual scrutiny without contextual considerations is however incomplete. Signs are not independent of their contexts (Martin & Rose 2003:1), which in this study would be authors, audiences and the spaces in which they are placed.

### The symbiotic relationship between sign producers and consumers

There is a synergetic relationship between these players that dictates what is seen or read and how it is interpreted. Hence, texts should be coherent, written or spoken, interpretable and meaningfully reader-immersed (Lou 2009:43) because they deal solely with meaning transmission from authors to readers (Malinowski 2009) as they are bound by contexts and reveal language use, while contexts reveal social relations (Hasan 1995:186) and issues. One question though is, do these factors take into consideration the effects (if any) of the three landscaping processes (the authorship, readership and connotation of signs) on social identity?

**FIGURE 3:**
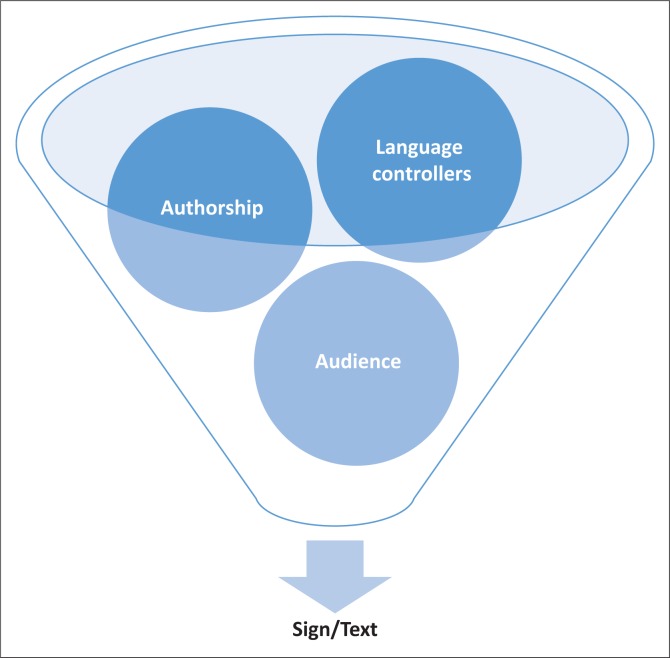
A graphic representation of the symbiotic relationship between sign producers and consumers.

Figure 3 reveals that each of these contributors is expected to have a mutual understanding of the information (texts and signs) which are specifically publicly placed. There is thus a symbiotic dependence on meaning by all contributors – authors, language controllers and the audience (Collins & Slembrouck 2007; Kallen 2009:274) with regard to the produced text. This consequently has a major impact on the interpretation of texts or signs by the audience as well as their reactions to the texts – reactions as a result of the impact of the text on their identity. Therefore, one of these categories of sign influencers cannot exist without the other. The symbiotic relationship of all contributors is thus of importance to this study as they are all intertwined and have a similar goal, which is to deliver meaning-bearing messages via the communicative modes of society. It also enables one to identify the cycle of text production, examination and the stance of the government or language controllers on pertinent issues in society. Their synergetic interactions thus present these three categories of sign influencers (authors, audience and language controllers) with different roles in the text and its connotation (Adekunle 2018).

### People with disabilities

**FIGURE 4:**
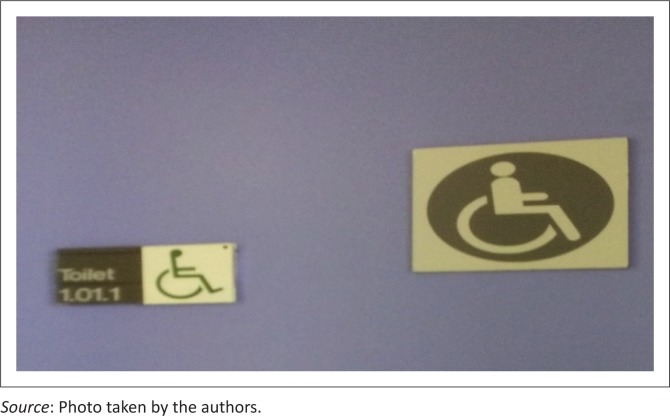
Toilet sections for people with disabilities.

Another noticeable section was the toilet space for people with disabilities (see Figure 4). One may easily assume that the authors of the signage were only being sensitive by allocating a separate and more expansive toilet space for users with disabilities. This is, however, in response to legal requirements (as specified in South African National Standard 2011:21–22). The impression is simply inferred by the affixed symbol of the man on a wheelchair and it connotes that only people within this category may use the specified toilet. This again is an example of the interwoven symbiotic relationship of contributors with a specific purpose: conveying meaning-bearing messages to target audiences.

This action on the part of the management of the institution indicates an understanding that people with disabilities might need a special and more expansive toilet space. By so doing, students with physical disabilities (or any disability) who might need to use the toilet are not deprived of the required space and facility (Mitra 2006). This, according to Nussbaum (2006), is justice for people with disabilities, which comprises special provisions that will enable them to have a gracious and convenient life on campus. However, the acknowledgement of students with disabilities should, according to Nussbaum (2006), be socially accepted and benefits should be equally and objectively disseminated. This cannot be said of the unavailability of gender specifications on the toilet space that has been provided for users with disabilities.

### Shortcomings of representations

This finding confirms Hart’s (2011:2) argument that inasmuch as group differences may help to identify forms of unfairness or discrimination, they may also insufficiently detect shortcomings. This highlights some of the shortcomings of the representation of group identities, which are grossly unequal. This finding (as observed on the symbols that were placed on the toilet doors) is a notable instance of the ‘degenderisation of disability’. It was noted in this study that even though the toilets of the able-bodied have gender specifications, those of people with disabilities lack an acknowledgement of gender identity. It is in fact connoted as ‘open for all people with disabilities’, without paying much attention to the gender differences of its users. This may also imply that the university accepts the common perception (as expressed by CEDAW 1989) in South African society with respect to prejudicing people with disabilities. This confirms Mutanga’s (2017:150) findings related to experiences of students with disabilities in South African higher education institutions which states that students with disabilities encounter diverse challenges in higher education institutions because of a lack of policy. Such a statement explains that this issue transcends universities’ stances to policies of both the institutions and government, especially in terms of universal access, spatial planning and existing policies that accommodate all the users of the space. It may also connote existing perceptions around sexual orientation and disability as maintained by Women with Disabilities Australia (n.d.), that people with disabilities are treated collectively as though they have no gender fluidity or sexual orientation. This genderless and collective treatment, according to Research Brief on Disability and Equality in South Africa (2017:1), may indeed birth or endorse the prevailing repudiation and exemption of people with disabilities and people of different sexual orientations in the affected society.

**FIGURE 5:**
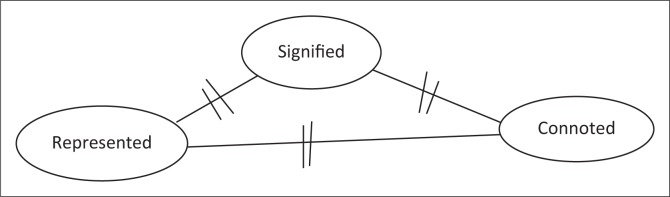
The conflict in communication.

The issue of language dominance is therefore of significance in this study and is explored via the identification of some gaps in language use and practice at the selected universities. A perception exists that discourse reveals ideological and power dynamics by means of studying texts that may reflect language inequality, racial segregation, ethnic differences and gender as well as sexuality issues, which explain people, their actions, reasons for those actions and society (Fairclough & Wodak 1997:258). There is therefore a conflict (Figure 5) between what a sign signifies, represents and connotes as a result of the language displayed, approved and used at both institutions.

These observations, among other things, could be termed as a prejudice: a form of inequality that is silently allowed to prevail, even though it is probably to the detriment (for instance – sexual harassment and undue exposure, among others) of users with disabilities who are all, regardless of gender and sexual differences, expected to share the same private space (students, staff and visitors alike). There are indeed issues relating to how the world is convolutedly expressed and linked by diverse ways of conception and understanding (Foucault 1972) and discourses such as these aid in describing the operational levels and semantics of communicative codes, which are also not devoid of hierarchical constructs that often reflect power dynamics.

Furthermore, the traditional disability symbol is a universal one, but this is also contested as it is not representative of all disabilities. Figure 4 may therefore also suggest an acknowledgement of only one of several other disabilities such as the non-visible disabilities (psychosocial, visual, hearing, learning and medical, among others), which form the majority of disabilities. The notion of reasonable accommodation needs to be further explored as a possible solution to the exclusion of certain groups of people, while also considering the provisions of the ninth section of the Constitution of the Republic of South Africa (Research Brief on Disability and Equality in South Africa 2017:7) as well as section 9 of the *Disability Discrimination Act* (Commonwealth Consolidated Acts 1992), among other formulated disability Acts across the globe. This emphasises the importance of environmental convenience and the removal of hurdles that may prevent an adequate accommodation of people with disabilities in society. Findings revealed the position of the universities on the bias of viewing all students with disabilities (or users of the space) as a collective entity that can use the same toilet space, irrespective of their gender or sexual identities, while able-bodied students have the ‘male’ or ‘female’ (and ‘gender-neutral’ – at University A) toilet space classifications. People with disabilities do not have the choice of a toilet based on their gender or sexuality. This could be construed as discriminatory. It contravenes the constitutional rights of that space for users (students, staff and visitors alike) who may have any form of disability.

### Implications of partial representations

The provision of one toilet for both men and women with disabilities at the institutions is entrenching deep-seated misconceptions about people with disabilities, which impedes the much-acclaimed inclusivity (Strnadova, Hájková & Květoňová 2015:1080) that South African universities preach. The inconsideration of the gender identities of people with disabilities is thus a subtle way of degenderising people with disabilities at the institutions by acknowledging them as a collective entity. Likewise, it is a means of re-conceptualising a misconception about people with disabilities, that is, ‘it is not important if people with disabilities share the same toilet, regardless of their gender identities – men, women, homosexual, transsexual, transgender, among others – at least they all have disabilities’.

Some themes on discursive silence in terms of some observed social issues such as gender identity, sexuality and the degenderisation of people with disabilities were also realised in the study. Notable levels of metaphorical silence with regard to these social phenomena were identified, especially with regard to the provision of services to people with disabilities and the acknowledgement of gender identities at the universities. These findings confirm Moletsane et al.’s (2004:61) assertion that the modal resources used in public spaces mostly do not, in practicality, speak to the existing linguistic and cultural diversity in the spaces. While there seems to be no progress in one university, as supported by the lack of posters and signage on the topic under investigation, there is some progress in the other university, although not sufficiently. Findings such as these should provoke queries with regard to assessing the impact of the inadequate and unequal reflection of sexual and gender identities of both the able bodied and people with disabilities. This is such that one begins to evaluate the perceptions around sexual orientation and disability and how daily linguistic practices also help to perpetuate subtle acts of divisiveness and labelling.

This, among other grave consequences, may lead to feelings of undue exposure and sexual harassment. Howell and Lazarus (2003) maintain that in order to deal with issues such as these in South Africa’s higher education institutions, there must be more focus on student diversity and other challenges (such as degenderisation). Fitchett (2015) expresses that work (that focuses on acknowledgement and access for students with disabilities) is currently in place in higher education institutions. The authors thus opine that the re-imagining of spaces and LL, by considering universal access as a way that welcomes and includes gender fluidity and the diverse abilities of all users of environments (be the user a student, staff member or visitor to campus) is not only pivotal to students’ integration in higher education, but is also indicative of continuous development, as well as the awareness and acknowledgement of the existing diversity within spaces.

Methodologically, CDA was effective in the detection of hidden and obvious themes in signage, while MDA enhanced the interpretation of the links between texts and images used dependently and independently, as well as the specific linguistic choices such as drawings, graphs and cartoons. Although this study advocates a change of scenario, especially with the use of these bi-pronged theoretical frameworks which also doubled as the methods of collection and analysis, one can only hope for change. Knowing that CDA for instance has been variously described as a form of critiquing that merely attempts to create change, Jäger and Maier (2009:36) argue that is not an ‘absolute truth’. Change creation in one place may be different from that of another; and issues regarding inequality and subordination are usually relative, depending on the society, the language as well as the perceptions and stances of the people living in that community.

## Conclusion

It can be concluded that the study of LLs is significant in the detection of power-related issues which oftentimes form part of the silent discourses prevalent in texts, their interactions in society and the medium of transmission to the readership. These findings may then be viewed from the perspective of the services (for instance, toilet spaces) rendered, the population in that given space and the supporting policies behind those structures, as opposed to the actual beneficiaries of those services. The degenderisation of disability and the little or no acknowledgement of gender differences, as observed on the toilet doors of the institutions, may unfortunately only be a reflector of other discriminatory and unequal measures with which people or processes are maintained in society. However, this cannot be used to make such generalisations because of limited data and the fact that only 2 out of 26 South African universities were examined. Moreover, limited data (as pertaining this subject area) were found and collected during the course of the examination of the existing LLs at both universities. It is thus not the researcher’s intention to generalise findings across all universities. This research can, however, serve as a pilot study.

On the other hand, this is a considerably complex terrain. It is also expected that there are diverse forms of barriers which may hinder or slow the pace of these developments. Some of these barriers may be linked to policy implications (institutional and national), the possibly lengthy processes of approval and the implementation of a more accommodating policy and universal design, as well as controversies that may arise from this sort of inclusion. Another prominent factor is the fact that there exist many social groups in society which may inadvertently feel or be excluded while certain others are being included. For instance, in having non-genderised toilet and bathroom areas in a university residence context, one may easily exclude certain religious groups that do not believe in shared ablution areas, just as in the case with toilets and bathrooms that are non-genderised for people with disabilities. The question then may thus be: where do certain religious groups fit in? As valid as the reasoning is, this present study however, only focuses on the accommodation of sexual orientation and disabilities via displayed LLs at the universities. The researchers opine that if able-bodied people on campus are allowed the luxury of using an appropriate gender-based toilet (acknowledging their well-deserved privacy and dignity), their counterparts with disabilities should also be accorded an equal right.

## Recommendations

An evaluation of this study’s findings provides an understanding that some changes cannot be expected overnight because there are multiple stakeholders engaged in any process of change. This is definitely an ongoing process of transformation in public spaces and perhaps some moves are in progress in ensuring these changes. The researchers will however highlight a few recommendations in this section based on the findings of the study. The recommendations discussed further are proffered towards ensuring an all-embracing and integrating academic space for students, staff and/or visitors at the universities.

Having noted that people with disabilities, under the South African Constitution, have a right to be treated equally, the management of universities should place some focus on the welfare of this group. Both able-bodied and students with disabilities are entitled to equal rights in their learning spaces. It is imperative to inquire about the well-being of students with disabilities in higher education institutions, after which a proposal should be put in place for the provision of an inclusive environment. It is thus of immense importance that there are equal and beneficial provisions of services to all students at the universities. Public and private organisations need to view this as an issue of identity, as well as societal perceptions about people with disabilities and their rights, which should be on an equal level with their able-bodied counterparts. Similarly, universities may need to be more forthright about their positions on sexuality, especially in relation to the law of the land where they are instituted. This is a step towards ensuring the appreciation and/or approval of all students, regardless of their sexual orientation and physical abilities or disabilities. Institutions of learning may also curb the widespread practice of degenderising people with disabilities through signage by making provisions for an all-encompassing policy and facility that will consider everyone (able-bodied and people with disabilities) equally. This will hopefully enhance the creation of an inclusive learning space or environment for all students.

From this policy perspective, more action must be taken to ensure a synchronisation of policy and the LLs, which are mostly the first sets of information that the audience is exposed to. For instance, both explored universities have standardised policies on disability and sexual orientations, which are accommodating and impressive. Issues of sensitivity and intentional inclusion without hypocrisy with regard to sexuality should therefore be considered by authors when planning LLs. This is because the signs that are expressed by means of the media are essential as they have an impact on the way the audiences of those signs perceive and comprehend them (Lacey 1998:35), themselves within the space and society at large. More importantly, the management of institutions should ensure that displayed signage reflects both the mandates of the nation’s policies, as well as the represented groups on campus. This will further assist in facilitating a balanced correlation between the policy of the institution and that of the nation, as well as the institution’s practice, while moving further towards ensuring inclusivity. These issues cannot be ignored as they play a significant role in human and social identities. It is thus vital to ensure that the modal resources (texts, signs, symbols) used on campuses are reflective of the readership that occupy the space. That is, everyone who uses the space must be considered in its spatial and language practices.

As findings revealed that, besides the collected data (discussed herein), no other mention was made of these groups in the public or private spaces (billboards or posters) of both universities, it is therefore important that there are avenues that may enable effective and productive interactions and engagements between all students (physically-abled, people with disabilities and gender identities) on campus. The researchers suggest that an attempt be made to involve people with disabilities in appropriate social engagements and interactions in a bid to have an adequate understanding of the challenges they may be encountering and the possible actions to take. This does not only impede exclusion, it also nurtures a warm and productive learning space where students feel welcome, acknowledged and are motivated towards active citizenry. This would not be a privilege; it is in fact their right.

## Possible areas of research focus

An examination of the opinions of students with disabilities is imperative in a bid to enhance inclusive practices in South Africa’s institutions of higher learning It would be crucial to understand their perspectives on the issues of representation and acceptance, with more emphasis on the modes to which they are exposed in their learning spaces. Such research could assist the government and management of the institutions in effecting change where necessary, while also affording authors sufficient knowledge of how displayed information possibly impacts the users of the space.

Research into the creation of a more inclusive environment for people with disabilities and people of diverse sexual orientations is necessary. An exploration of this via LLs and existing resources, among other approaches, may be a push towards the possibility of having universally designed spaces that may not necessarily provide solutions (at least not immediately), but a reasonable accommodation in South Africa’s institutions of higher learning and SA as a whole. This is crucial if spaces are to be made available and beneficial to all.
